# The Lighting of the Chiswick Hospital

**Published:** 1913-05-24

**Authors:** 


*To the Editor of* THE HOSPITAL.

### The Lighting of the Chiswick Hospital.

*To the Editor of* THE HOSPITAL.

SIR,—We were pleased to see in your issue of May 3 an article describing the lighting of the Chiswick Cottage Hospital, and were extremely interested in your contributor's reference to the suitability of the British Thomson-Houston Company's "Eye-Rest" system of lighting for hospital wards. We think everyone will agree it is very desirable that all powerful light sources should be adequately screened from the view of the patients, as is the case with our "Eye-Rest" system.

With reference to the statement in the article that the general illumination of the wards was provided by a specially-designed fitting of the "Eye-Rest" lamps of the Thomson-Houston Company, may we take this opportunity to point out that the specially designed indirect lighting fittings are known under the registered name of the "Eye-Rest" system, and that the fittings themselves contain "Mazda" drawn-wire electric lamps, both fittings and lamps being supplied by the British Thomson-Houston Company? It is the system of lighting which is known as the "Eye-Rest" system, the bulbs used being the "Mazda" drawn-wire incandescent lamps, which, owing to their high quality, make them particularly suitable for this class of illumination.
—Yours faithfully,

The British Thomson-Houston Company, Limited.

Mazda House,
77 Upper Thames Street, London, E.C.,
May 16, 1913.

